# Impact of amyloid and tau positivity on longitudinal brain atrophy in cognitively normal individuals

**DOI:** 10.1186/s13195-024-01450-7

**Published:** 2024-04-10

**Authors:** Motonobu Fujishima, Yohei Kawasaki, Toshiharu Mitsuhashi, Hiroshi Matsuda, for the Alzheimer’s Disease Neuroimaging Initiative

**Affiliations:** 1 Department of Radiology, Kumagaya General Hospital, 4-5-1 Nakanishi, Kumagaya, 360-8567 Japan; 2 Department of Biostatistics, Graduate School of Medicine, Saitama Medical University, 38 Morohongo, Moroyama, 350-0495 Japan; 3 Biostatistics Section, Clinical Research Center, Chiba University Hospital, 1-8-1 Inohana, Chuo-Ku, Chiba, 260-8670 Japan; 4 Center for Innovative Clinical Medicine, Okayama University Hospital, 2-5-1 Shikata-Cho, Kita-Ku, Okayama, 700-8558 Japan; 5 Department of Biofunctional Imaging, Fukushima Medical University, 1 Hikariga-Oka, Fukushima, 960-1295 Japan; 6 Drug Discovery and Cyclotron Research Center, Southern Tohoku Research Institute for Neuroscience, 7-61-2 Yatsuyamada, Koriyama, 963-8052 Japan

**Keywords:** Preclinical, Alzheimer’s disease, Longitudinal MRI, Tau, Amyloid-β

## Abstract

**Background:**

Individuals on the preclinical Alzheimer's continuum, particularly those with both amyloid and tau positivity (A + T +), display a rapid cognitive decline and elevated disease progression risk. However, limited studies exist on brain atrophy trajectories within this continuum over extended periods.

**Methods:**

This study involved 367 ADNI participants grouped based on combinations of amyloid and tau statuses determined through cerebrospinal fluid tests. Using longitudinal MRI scans, brain atrophy was determined according to the whole brain, lateral ventricle, and hippocampal volumes and cortical thickness in AD-signature regions. Cognitive performance was evaluated with the Preclinical Alzheimer's Cognitive Composite (PACC). A generalized linear mixed-effects model was used to examine group × time interactions for these measures. In addition, progression risks to mild cognitive impairment (MCI) or dementia were compared among the groups using Cox proportional hazards models.

**Results:**

A total of 367 participants (48 A + T + , 86 A + T − , 63 A − T + , and 170 A − T − ; mean age 73.8 years, mean follow-up 5.1 years, and 47.4% men) were included. For the lateral ventricle and PACC score, the A + T − and A + T + groups demonstrated statistically significantly greater volume expansion and cognitive decline over time than the A − T − group (lateral ventricle: β = 0.757 cm^3^/year [95% confidence interval 0.463 to 1.050], *P* < .001 for A + T − , and β = 0.889 cm^3^/year [0.523 to 1.255], *P* < .001 for A + T + ; PACC: β =  − 0.19 /year [− 0.36 to − 0.02], *P* = .029 for A + T − , and β =  − 0.59 /year [− 0.80 to − 0.37], *P* < .001 for A + T +). Notably, the A + T + group exhibited additional brain atrophy including the whole brain (β =  − 2.782 cm^3^/year [− 4.060 to − 1.504], *P* < .001), hippocampus (β =  − 0.057 cm^3^/year [− 0.085 to − 0.029], *P* < .001), and AD-signature regions (β =  − 0.02 mm/year [− 0.03 to − 0.01], *P* < .001). Cox proportional hazards models suggested an increased risk of progressing to MCI or dementia in the A + T + group versus the A − T − group (adjusted hazard ratio = 3.35 [1.76 to 6.39]).

**Conclusions:**

In cognitively normal individuals, A + T + compounds brain atrophy and cognitive deterioration, amplifying the likelihood of disease progression. Therapeutic interventions targeting A + T + individuals could be pivotal in curbing brain atrophy, cognitive decline, and disease progression.

**Supplementary Information:**

The online version contains supplementary material available at 10.1186/s13195-024-01450-7.

## Background

From 2022–2023, the results of phase III clinical trials of the anti-amyloid-beta (Aβ) monoclonal antibodies lecanemab and donanemab for Alzheimer's disease (AD) were published [1, 2], with the former approved by the U.S. Food and Drug Administration (FDA) and the latter under review for approval. These disease-modifying drugs were designed based on the amyloid cascade hypothesis in AD [3]. The hypothesis is that Aβ aggregates and forms fibrils outside of neurons in the cerebral cortex; then, hyperphosphorylated tau aggregates and forms neurofibrillary tangles within neurons, which spread to the cortex, causing progressive synaptic dysfunction and neurodegeneration. Ultimately, this process results in cognitive decline and the development of dementia. The target population for treatment with these disease-modifying drugs is patients with mild cognitive impairment (MCI) or mild dementia but not patients with advanced dementia.

More recently, the therapeutic focus of disease-modifying drugs has been directed to elderly population in earlier stages of AD. For example, the AHEAD 3–45 trial (NCT04468659) [4] is a clinical trial of lecanemab for asymptomatic elderly population with amyloid positivity (A +); A + corresponds to the condition defined as the Alzheimer’s continuum in the 2018 National Institute on Aging and Alzheimer's Association (NIA-AA) research framework [5]. The Alzheimer's continuum based on the NIA-AA criteria includes three categories: Amyloid and tau positivity (A + T +) is defined as “Alzheimer’s disease”, and amyloid positivity and tau negativity (A + T −) is defined as “Alzheimer's pathologic change” if neurodegeneration is not present or “Alzheimer's and concomitant suspected non-Alzheimer's pathologic change” if neurodegeneration is present. Among these categories, preclinical AD (A + T +) is receiving more attention because it is associated with faster cognitive decline and a higher risk of progression to MCI than A − T − and A + T − [6, 7].

Determining how long and to what extent brain atrophy progresses in the elderly population on the preclinical Alzheimer's continuum would contribute to a better understanding of the pathophysiology of neurodegeneration and a more accurate interpretation of the results of clinical trials of individuals on the Alzheimer's continuum. Although several research groups have already reported accelerated cerebral atrophy in subjects with A + T + compared with subjects with A − T − [8–10], the average observation period for these studies was short at approximately two years. Longitudinal structural magnetic resonance imaging (MRI) can estimate the extent of brain atrophy over time before the first clinical manifestations of AD appear [11], but few studies with long follow-up periods (more than 4–5 years) have reported how long and to what extent brain atrophy is accelerated in elderly individuals on the preclinical Alzheimer's continuum.

The hypothesis of this study was that the elderly population on the preclinical Alzheimer's continuum have accelerated brain atrophy compared with those with amyloid and tau negativity (A − T −). Longitudinal analysis of structural MRI and cognitive performance and survival analysis of progression to MCI or dementia were performed on four groups consisting of both positive and negative combinations of A and T biomarkers, which are neuropathological features of AD, using long-term follow-up data.

## Methods

### Participants

The Alzheimer’s Disease Neuroimaging Initiative (ADNI) was launched in 2003 as a public–private partnership, led by principal investigator Michael W. Weiner, MD. The primary goal of ADNI has been to test whether serial MRI, PET, other biological markers, and clinical and neuropsychological assessment can be combined to measure the progression of MCI and early AD.

**Fig. 1:**
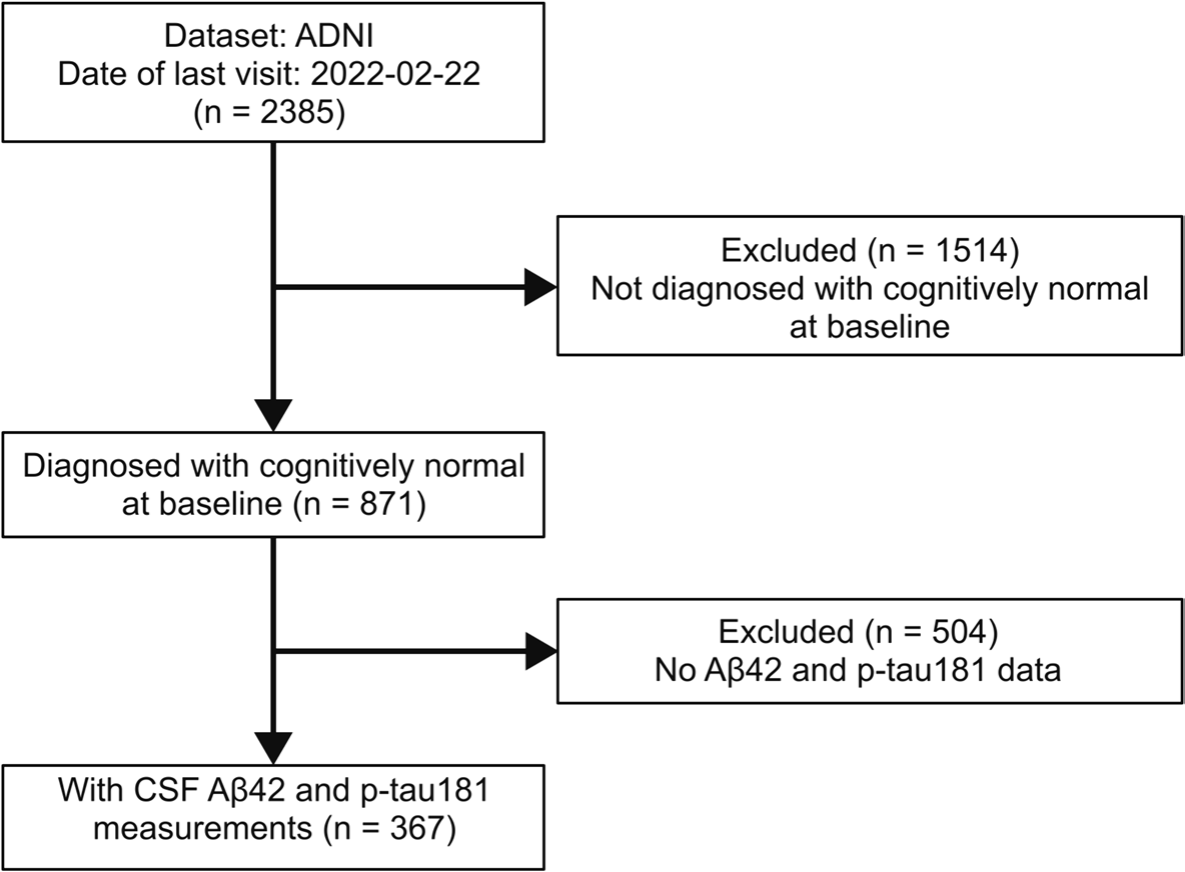
Flow diagram of the participant selection from the ADNI dataset. Abbreviations: Aβ42 = amyloid-beta 42; ADNI = Alzheimer's Disease Neuroimaging Initiative; CSF = cerebrospinal fluid; p-tau181 = phosphorylated tau 181

In the present study, 367 participants who had cerebrospinal fluid (CSF) Aβ42 and phosphorylated tau 181 (p-tau181) data were drawn from the ADNI 1, 2, GO, and 3 datasets. We used the following data with the last visit on 2022–02-22 from the ADNI website: ADNIMERGE.csv. All the participants in the present study were diagnosed as cognitively normal at baseline. They had baseline scores of 24 to 30 on the Mini-Mental State Examination (MMSE) [12] and global scores of 0 on the Clinical Dementia Rating (CDR) [13]. Their scores on the Wechsler Memory Scale-Revised (WMS-R) Logical Memory II [14] were based on the number of years of education: ≥ 3 for education of 0 to 7 years, ≥ 5 for 8 to 15 years, and ≥ 9 for ≥ 16 years. If a participant had a significant subjective memory concern, it was reported by the participant, study partner, or clinician. All participants were followed up six months after baseline, one year, and every year beyond. A flow diagram showing the process of participant selection for this study is presented in Fig. 1.

### Diagnostic group assignment based on amyloid and tau positivity/negativity by CSF biomarker measurements

CSF concentrations of Aβ42 and p-tau181 were measured with the Elecsys immunoassays using the cobas e601 analyzer (Roche Diagnostics GmbH, Mannheim, Germany) [15, 16]. In the present study, the cutoff values for CSF concentrations of Aβ42 and p-tau181 were defined as 981 pg/mL based on 18F-florbetapir PET and 24.3 pg/mL based on 18F-flortaucipir PET, respectively [17]; namely, participants with Aβ42 < 981 pg/mL were classified as amyloid positive (A +). Participants with p-tau181 > 24.3 pg/mL were classified as tau positive (T +).

### MRI acquisition and processing

#### Image registration of serial T1-weighted (T1w) MRI scans

**Fig. 2:**
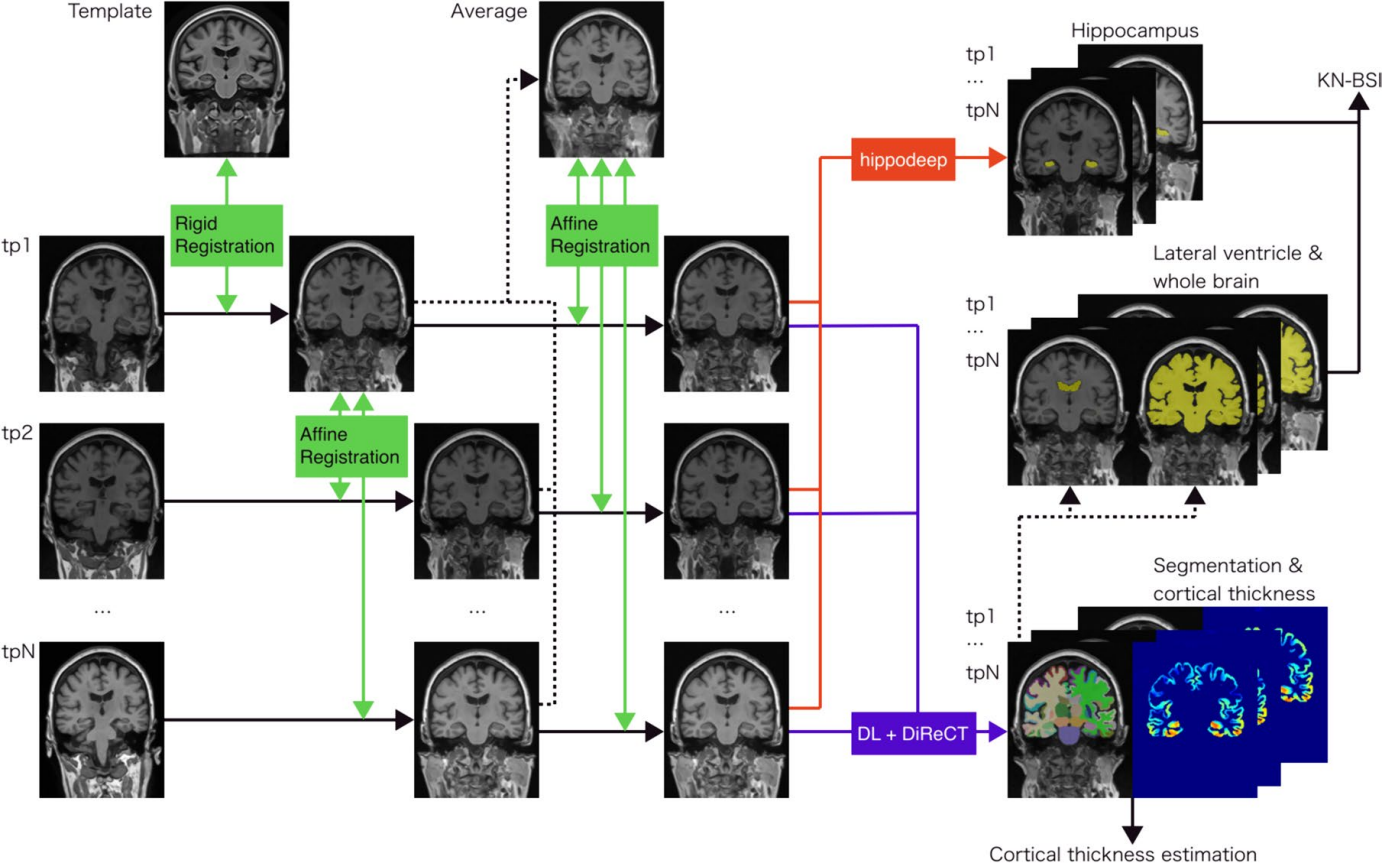
Image processing pipeline for the KN-BSI and cortical thickness computation. First, T1w scans at each timepoint were affinely registered to the baseline scan that had been rigidly registered to the ADNI template. Second, an averaged image was generated from the affinely registered scans at each timepoint. Third, T1w scans at each timepoint were affinely registered to the averaged image. DL + DiReCT was applied to the affinely registered image at each time point for segmentation and cortical thickness estimation. Segmentations for the whole brain and lateral ventricle were generated from the resultant images from DL + DiReCT. Hippocampal segmentations were generated using hippodeep. Finally, KN-BSI was computed for longitudinal volume changes in the whole brain, lateral ventricle, and hippocampus using the affinely registered images and segmentations. Abbreviations: KN-BSI, k-means normalized boundary shift integral; DL + DiReCT, deep learning-based neuroanatomical segmentation + diffeomorphic registration-based cortical thickness; tpN, Nth timepoint

All the participants had undergone more than one T1w MRI scan on a 1.5-T or 3-T scanner. The imaging protocols were described by Jack et al. [18]. All the serial T1w MRI scans were corrected for intensity inhomogeneity with N4ITK [19] following noise reduction with non-local means [20]. The baseline scan was then rigidly registered to the ADNI template with NiftyReg [21, 22]. The ADNI template was created from T1w MRI scans of 52 cognitively normal participants with antsMultivariateTemplateConstruction.sh script implemented in ANTs [23, 24]. All the follow-up scans were affinely registered to the baseline scan rigidly registered to the template with NiftyReg. The averaged image was created from the registered serial scans. Finally, all the serial scans were affinely registered to the averaged scan with NiftyReg. The image registration described here is depicted in Fig. 2.

### Computation of cerebral cortical thickness

Regional cerebral thickness was computed from the serial scans affinely registered to the averaged image above with DL + DiReCT [25]. DL + DiReCT is a method combining deep learning (DL)-based neuroanatomical segmentation and cortex parcellation with diffeomorphic registration-based cortical thickness (DiReCT) measurement [26]. We computed the voxel-weighted average of the mean cortical thickness within the bilateral entorhinal, fusiform, inferior temporal, and middle temporal regions defined by the Desikan-Killiany parcellations [27]. These regions were derived from the AD-signature regions of interest [28]. The cortical thickness computation described here is depicted in Fig. 2.

### Computation of k-means normalized boundary shift integral (KN-BSI)

To compute volume changes in the hippocampus, lateral ventricle, and whole brain on individual serial MRI scans, KN-BSI [29] was adopted. The hippocampal labels were automatically segmented with hippodeep [30] from the registered serial scans (Fig. 1). The lateral ventricle and whole brain labels were extracted from the segmentations created by DL + DiReCT above (Fig. 2). Symmetric differential bias correction (DBC) [31, 32] was applied to the serial scans to correct the differences in intensity inhomogeneity among the serial scans. A median filter with a radius of five voxels for DBC was adopted with the original reference [32]. Volume changes in the lateral ventricle and whole brain were computed with normal KN-BSI from the DBC-corrected scans. In contrast, volume changes in the hippocampus were computed with a double intensity-window KN-BSI to capture boundary shift at both the hippocampus-CSF border and the hippocampus-white matter border [33] from the DBC-corrected scans. The volume at each timepoint was calculated by subtracting the volume change between the baseline and each timepoint as calculated by KN-BSI from the volume at the baseline.

### Cognitive assessment

We adapted the Preclinical Alzheimer Cognitive Composite (PACC) to assess subtle cognitive changes for cognitively normal participants with A + and/or T + status. The PACC in the current study was a baseline standardized z score composite of the Delayed Word Recall score from the Alzheimer's Disease Assessment Scale—Cognitive Subscale, the Delayed Recall score on the Logical Memory IIA subtest from the WMS-R, the MMSE, and log-transformed Trail-Making Test B Time to Completion [34]. Note that the PACC score decreases with worse cognitive performance.

### Statistical analyses

Descriptive statistics were calculated for the data obtained in this study. They are presented as means and standard deviations for continuous quantities and as frequencies and proportions for categorical variables. The CSF concentrations of Aβ42 < 200 pg/mL and > 1,700 pg/mL were set as 200 pg/mL and 1,700 pg/mL, respectively, for the statistics because CSF Aß42 had the lower and upper technical limits of measurement of < 200 mg/mL and 1,700 pg/mL, respectively. Similarly, the CSF concentrations of tau < 80 pg/mL and p-tau181 < 8 pg/mL were set as 80 pg/mL and 8 pg/mL, respectively, for the statistics because CSF tau and CSF p-tau181 had lower technical limits of the measurement of < 80 mg/mL and 8 pg/mL, respectively.

**Fig. 3:**
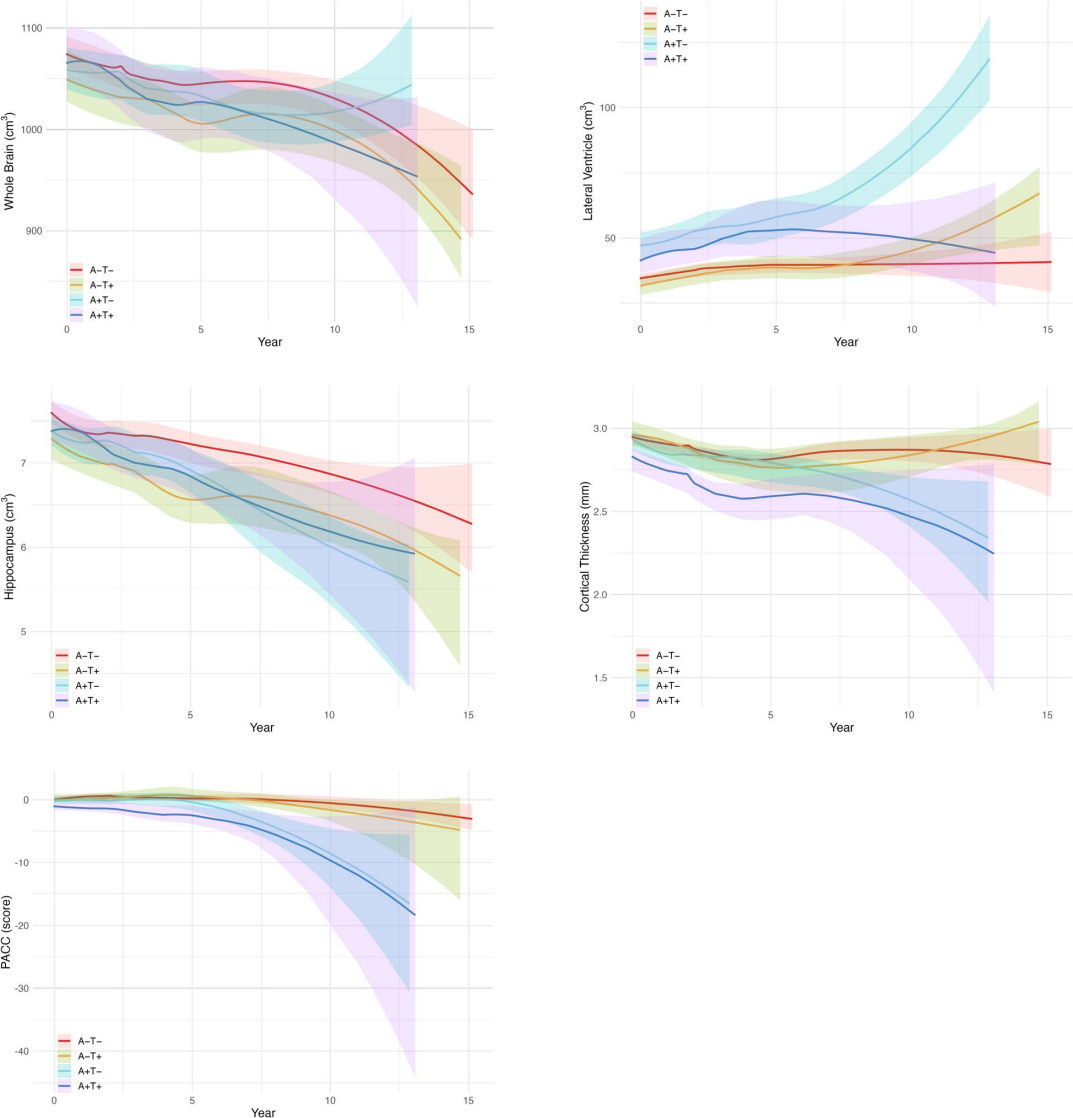
Mean measurement trajectories and 95% confidence bands using LOESS for each group classified by amyloid and tau positivity/negativity. Abbreviations: A −  = amyloid negative; A +  = amyloid positive; LOESS = locally estimated scatterplot smoothing; PACC = Preclinical Alzheimer Cognitive Composite; T −  = tau negative; T +  = tau positive

Outcome changes for each of the four groups assigned by amyloid and tau positivity/negativity were analyzed using a generalized linear mixed-effects model. The following five variables were selected as response variables: whole brain volume, lateral ventricular volume, hippocampal volume, cortical thickness in the AD-signature regions of interest, and the PACC score. The linearity of the relationships between outcome and time was assessed by locally estimated scatterplot smoothing (LOESS) [35]. Figure 3 shows the LOESS plots for the response variables in the four groups. In addition, 95% confidence bands calculated by bootstrapping with 10,000 resamples were added to the graph. Based on the linear trends delineated by these plots, we determined to use a generalized linear mixed-effects model with a linear term for time of up to 7.5 years from the baseline as the main analysis. Models that included quadratic terms were also examined and compared using the Akaike Information Criterion (AIC) [36] to assess the goodness of fit of the data (Supplementary Table 1). Although the AIC results were better for the models that included quadratic terms, a linear model was adopted due to ease of interpretability. Due to the very small amount of data obtained after 7.5 years in this study, these data were excluded from the main analysis. Furthermore, two sensitivity analyses were conducted: First, a generalized linear mixed-effects model incorporating additional interactions between covariates and time was employed to examine these interactions. Second, a generalized linear mixed-effects model was used to analyze the data harmonized for variability between different MR scanners. The harmonization was applied to the MRI measurements using the longCombat package [37] in R version 4.2.1 (R Foundation for Statistical Computing, Vienna, Austria) to alleviate non-biological variations in data acquired from multiple MR scanners with magnetic field strengths of 1.5 T and 3 T across multiple research sites. The explanatory variables selected were the group (A − T − group as the reference), time (in years), the interaction term between the group and time, and candidate confounders (presence of subjective memory concerns, age at baseline, sex, number of years of education, and number of *APOE* ε4 alleles). For analyses where the response variable was either the whole brain volume, lateral ventricular volume, or hippocampal volume, intracranial volume at baseline from ADNIMERGE.csv was also included as a confounding factor. These confounding factors were determined in previous studies [38, 39]. Each of the mixed-effects model assumed a random intercept and random slope at the individual level, modeling that each measurement was nested in individuals. The overall group effect was tested using a likelihood ratio test comparing the full model to a reduced model, excluding the group variable. If the test was found to be significant, under the predictions calculated by this model, modeled means were calculated for each group and each year, and approximate curves were drawn using fractional polynomial regressions.

In the sub-analysis, the same analysis as the main analysis was performed using all data (i.e., including 7.5 years and beyond). Based on visual inspection of the LOESS plots (Fig. 3), we also included the quadratic term for time and interaction terms between the group and the quadratic term for time in the generalized linear mixed-effects model. As in the main analysis, the overall group effect was tested using a likelihood ratio test comparing the full model to a reduced model, excluding the group variable. In addition, the same analysis was performed on the data harmonized using longCombat for the MRI measurements as a sensitivity analysis.

Next, survival analysis was performed for conversion to MCI or dementia using data from the entire period. Kaplan–Meier curves were drawn for each group and compared by the log-rank test. The Cox proportional hazards model with the group as the explanatory variable was then used to calculate the hazard ratio and its 95% confidence interval for the A − T − group as a reference. First, the unadjusted model was applied. Second, the multivariable model was adjusted for baseline age, sex, number of years of education, *APOE* ε4 status, and presence of subjective memory concerns.

All tests were two-tailed, and *p*-values less than 0.05 were considered statistically significant. This study was an exploratory analysis; therefore, no alpha adjustment was made to control for Type 1 errors. Stata 18/MP (Stata Crop LLC, College Station, TX, USA) was used for the analysis. LOESS graphs were drawn using ggplot2 in R.

## Results

### Demographic characteristics

Among 367 participants in the study, the mean (standard deviation) age was 73.8 (5.9) years, and the observation period was 5.1 (3.4) years; there were 174 male (47.4%) and 193 female (52.6%) participants. Approximately 13.1% (48/367) of participants were classified as A + T + using CSF concentrations of Aβ42 and p-tau181, compared with 23.4% (86/367) as A + T − , 17.2% (63/367) as A − T + , and 46.3% (170/367) as A − T − . Further demographic characteristics are depicted in Table 1.

**Table 1:** Participant characteristics by amyloid and tau positivity/negativity classification

	A + T + (*n* = 48)	A + T − (*n* = 86)	A − T + (*n* = 63)	A − T − (*n* = 170)
**Baseline Characteristics**				
Age, mean (SD), y	76.3 (5.1)	73.4 (5.9)	75.8 (6.6)	72.6 (5.5)
No. of male (%)	21 (44)	40 (47)	28 (44)	85 (50)
Education, mean (SD), y	16.2 (2.5)	16.4 (2.7)	16.5 (2.7)	16.3 (2.6)
Ethnicity (%)				
Not Hispanic/Latino	47 (98)	84 (98)	61 (97)	160 (94)
Hispanic/Latino	1 (2)	2 (2)	1 (2)	8 (5)
Unknown	0 (0)	0 (0)	1 (2)	2 (1)
Race (%)				
American Indian/Alaskan native	0 (0)	1 (1)	0 (0)	0 (0)
Asian	0 (0)	1 (1)	1 (2)	2 (1)
Black	2 (4)	8 (9)	1 (2)	13 (8)
White	46 (96)	72 (84)	61 (97)	154 (91)
More than one race	0 (0)	4 (5)	0 (0)	1 (1)
Subjective memory concern (%)	13 (27)	17 (20)	17 (27)	48 (28)
*APOEε4* allele = 1 (%)	25 (52)	29 (34)	13 (21)	27 (16)
*APOEε4* allele = 2 (%)	2 (4)	6 (7)	0 (0)	1 (1)
PACC, mean (SD)	− 0.97 (2.41)	− 0.30 (2.80)	− 0.10 (2.72)	0.07 (2.33)
MMSE, mean (SD)	29.1 (1.2)	29.1 (1.1)	28.9 (1.3)	29.1 (1.1)
CDR-SB (%)				
0	43 (90)	76 (88)	58 (92)	161 (95)
0.5	4 (8)	10 (12)	5 (8)	9 (5)
1	1 (2)	0 (0)	0 (0)	0 (0)
CSF Aβ_42_, mean (SD), pg/mL	706.8 (177.8)^a^	689.9 (203.2)	1548.5 (226.8)^b^	1479.3 (230.3)^b^
CSF tau, mean (SD), pg/mL	346.5 (73.9)	173.1 (49.2)^c^	349.2 (69.1)	197.6 (39.9)
CSF p-tau_181_, mean (SD), pg/mL	35.5 (8.7)	16.0 (4.7)^d^	31.3 (6.8)	17.3 (3.5)
Whole brain volume, mean (SD), cm^3^	1061.949 (94.060)	1059.643 (102.777)	1045.114 (96.797)	1073.724 (109.641)
Ventricular volume, mean (SD), cm^3^	39.782 (19.863)	47.447 (24.799)	31.686 (14.950)	34.223 (14.294)
Hippocampal volume, mean (SD), cm^3^	7.360 (0.846)	7.395 (0.943)	7.291 (0.976)	7.607 (0.920)
Cortical thickness, mean (SD), mm	2.84 (0.25)	2.92 (0.28)	2.95 (0.26)	2.95 (0.21)
**Follow-up characteristics**				
Follow-up, mean (SD), y	4.4 (3.1)	4.5 (3.0)	5.6 (3.7)	5.5 (3.5)
Follow-up CSF measurements available (%)	32 (67)	44 (51)	29 (46)	107 (63)
Progression to amyloid-positive (%)	NA	NA	6 (10)	14 (8)
Progression to tau-positive (%)	NA	7 (8)	NA	10 (6)
Progression to dementia (%)	6 (12)	5 (6)	4 (6)	3 (2)

*Abbreviations*: *CDR-SB* Clinical Dementia Rating Sum of Boxes, *CSF* Cerebrospinal fluid, *MMSE* Mini-Mental State Examination, *NA* Not applicable, *PACC* Preclinical Alzheimer Cognitive Composite, *SD* Standard deviation

^a^Concentration of CSF Aβ_42_ < 200 pg/mL was set as 200 pg/mL for the statistics due to the lower technical limit of the measurement

^b^Concentration of CSF Aβ_42_ > 1700 pg/mL was set as 1700 pg/mL for the statistics due to the upper technical limit of the measurement

^c^Concentration of CSF tau < 80 pg/mL was set as 80 pg/mL for the statistics due to the lower technical limit of the measurement

^d^Concentration of CSF p-tau_181_ < 8 pg/mL was set as 8 pg/mL for the statistics due to the lower technical limit of the measurement

### Changes in longitudinal structural MRI measurements and PACC scores over time

#### Main analysis up to 7.5 years from the baseline

**Fig. 4:**
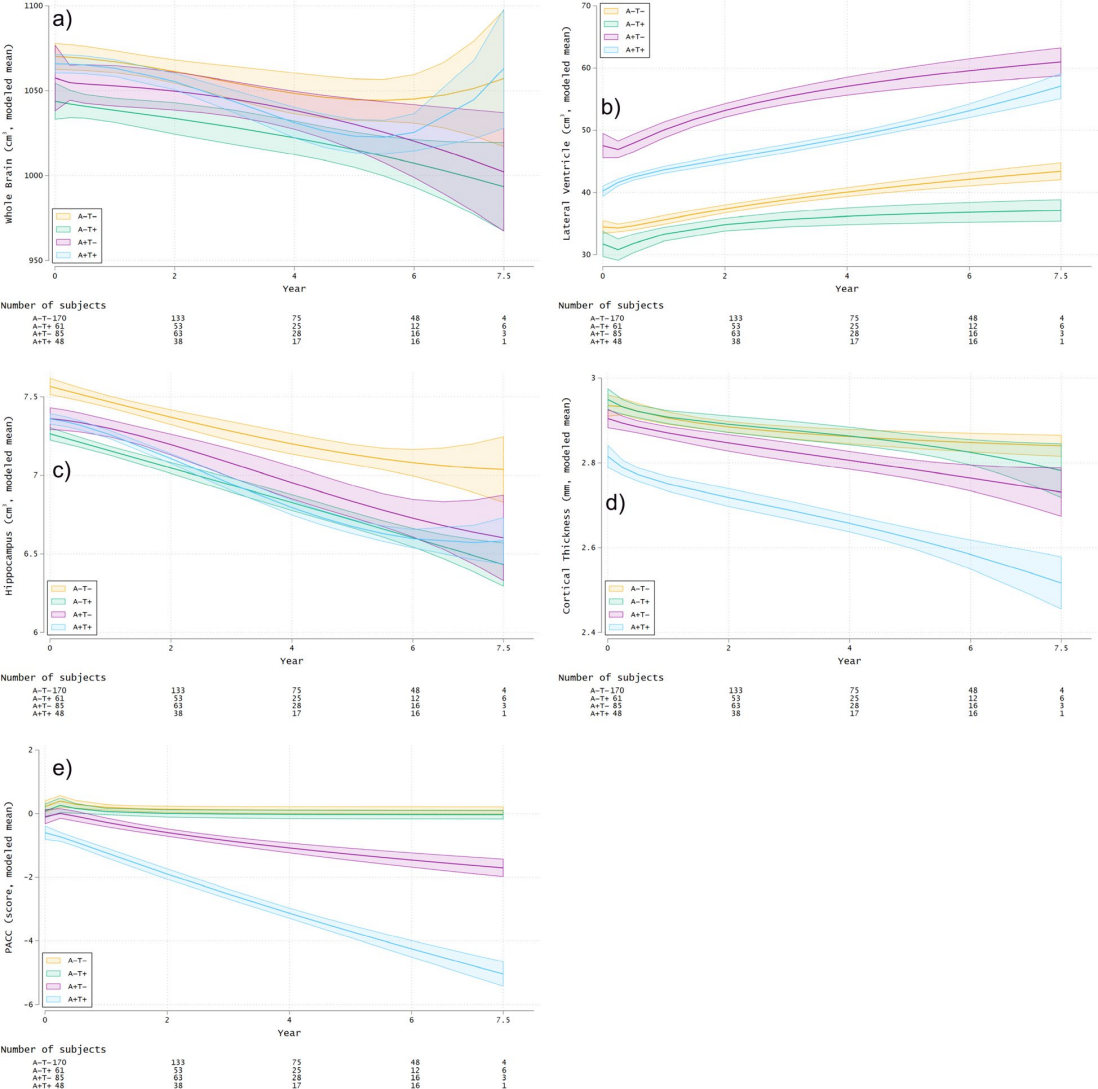
Changes in longitudinal MRI measurements and cognitive performance by amyloid and tau positivity/negativity classification over 7.5 years. **a**-**e** Trajectories of modeled mean profiles of whole brain volume, lateral ventricular volume, hippocampal volume, cortical thickness, and PACC scores based on a generalized linear mixed-effects model and 95% confidence bands. The models were controlled for baseline age, *APOE* ε4 status, sex, number of years of education, and baseline intracranial volume (only for the volumetric measures). Abbreviations: A −  = amyloid negative; A +  = amyloid positive; PACC = Preclinical Alzheimer Cognitive Composite; T −  = tau negative; T +  = tau positive

Figure 4 reveals modeled mean profiles for the volumes of the whole brain, lateral ventricle, and hippocampus, cortical thickness in the AD-signature regions of interest, and PACC scores in the four groups from baseline to 7.5 years based on a generalized linear mixed-effects model. Three cases (two A−T+ and one A+T−) had no data at baseline. This model was controlled for the covariates including baseline age, *APOE* ε4 status (0, 1, or 2), sex, number of years of education, and baseline intracranial volume (only for the volumetric measures). The likelihood ratio test for the group effect was significant for the whole brain (χ2(6) = 27.0; *P* < .001), lateral ventricle (χ2(6) = 78.9; *P* < .001), hippocampus (χ2(6) = 19.0; *P* = .004), cortical thickness (χ2(6) = 29.2; *P* < .001), and PACC score (χ2(6) = 32.8; *P* < .001). Since these likelihood ratio tests were significant, we determined that including group variables in the statistical model was necessary.

For the whole brain and hippocampal volume and cortical thickness, only the A+T+ group showed statistically significantly greater volume loss and cortical thinning over time than the A−T− group (β = −2.782 cm^3^/year, 95% confidence interval (CI) = −4.060 to −1.504, *P* < .001 for the whole brain volume, β = −0.057 cm^3^/year, 95% CI = −0.085 to −0.029, *P* < .001 for the hippocampal volume, and β = −0.02 mm/year, 95% CI = −0.03 to −0.01, *P* < .001 for the cortical thickness; Figure 4a, c, d, and Table 2). For the lateral ventricular volume, the A+T− and A+T+ groups showed statistically significantly greater volume expansion over time than the A−T− group (β = 0.757 cm^3^/year, 95% CI = 0.463 to 1.050, *P* < .001 for A+T− vs. A−T−, and β = 0.889 cm^3^/year, 95% CI = 0.523 to 1.255, *P* < .001 for A+T+ vs. A−T−; Figure 4b and Table 2). For the PACC score, the A+T+ and A+T− groups showed statistically significantly greater cognitive decline over time than the A−T− group (β = −0.19/year, 95% CI = −0.36 to −0.02, *P* = .029 for A+T− vs. A−T−, and β = −0.59/year, 95% CI = −0.80 to −0.37, *P* < .001 for A+T+ vs. A−T−; Figure 4e and Table 2). Furthermore, based on these 95% CIs, the A+T+ group showed statistically significantly greater decline in cognitive function over time than the A+T− group (Table 2). The A−T+ group did not show greater brain atrophy and cognitive decline than the A−T− group based on MRI measures and the PACC scores, respectively. The main effects (group differences at Time = 0) are shown in the Group row in Table 2. A consistent trend was also confirmed in the model with additional covariate and time interactions as a sensitivity analysis (Supplementary Table 2). In the model, significant associations were observed between the baseline age and longitudinal changes in the expansion of the lateral ventricles, cortical thinning, and lower PACC scores (Supplementary Table 2). In the sensitivity analysis of the data harmonized for MRI measurements using longCombat, the model yielded consistent results with those obtained from the analysis of non-harmonized data (Supplementary Table 3 and Supplementary Figure 1).

**Table 2:** Summary of a generalized linear mixed-effects model in the main analysis for serial structural MRI and cognitive performance measures from baseline to 7.5 years

	Whole brain			Lateral ventricle			Hippocampus		
	β	95% CI	*P*-value	β	95% CI	*P*-value	β	95% CI	*P*-value
		Lower, Upper			Lower, Upper			Lower, Upper	
Intercept	1353.323	1281.298, 1425.348	< .001	− 21.463	− 41.046, − 1.880	.032	11.707	10.577, 12.838	< .001
Age	− 4.239	− 5.108, − 3.370	< .001	0.767	0.532, 1.002	< .001	− 0.051	− 0.065, − 0.037	< .001
Male	16.673	4.900, 28.447	.006	− 1.893	− 5.077, 1.290	.244	0.102	− 0.083, 0.287	.280
Years of education	− 0.349	− 2.261, 1.563	.720	0.194	− 0.322, 0.710	.462	− 0.039	− 0.069, − 0.009	.012
*APOE* ε4 alleles	16.491	− 3.813, 36.795	.111	− 8.790	− 14.296, − 3.283	.002	0.083	− 0.236, 0.402	.609
Baseline ICV	0.541	0.504, 0.577	< .001	0.042	0.032, 0.052	< .001	0.003	0.002, 0.003	< .001
SMC	14.324	2.965, 25.682	.013	2.182	− 0.916, 5.280	.168	0.067	− 0.111, 0.245	.461
Group									
A − T +	5.763	− 8.256, 19.782	.420	− 3.720	− 8.059, 0.619	.093	− 0.028	− 0.248, 0.192	.802
A + T −	− 16.684	− 29.442, − 3.925	.010	13.526	9.590, 17.462	< .001	− 0.168	− 0.368, 0.033	.101
A + T +	− 2.018	− 18.274, 14.238	.808	4.370	− 0.609, 9.350	.085	− 0.055	− 0.310, 0.200	.673
Time	− 5.878	− 6.450, − 5.306	< .001	1.239	1.072, 1.406	< .001	− 0.091	− 0.103, − 0.078	< .001
Group × time									
A − T + × time	− 0.321	− 1.441, 0.800	.575	− 0.038	− 0.363, 0.287	.819	− 0.023	− 0.047, 0.002	.069
A + T − × time	− 0.524	− 1.548, 0.500	.316	0.757	0.463, 1.050	< .001	− 0.015	− 0.038, 0.007	.188
A + T + × time	− 2.782	− 4.060, − 1.504	< .001	0.889	0.523, 1.255	< .001	− 0.057	− 0.085, − 0.029	< .001
	Cortical thickness					PACC			
	β	95% CI		*P*-value		β	95% CI		*P*-value
		Lower, Upper					Lower, Upper		
Intercept	4.19	3.87, 4.51		< .001		6.65	3.52, 9.78		< .001
Age	− 0.02	− 0.02, − 0.01		< .001		− 0.13	− 0.17, − 0.09		< .001
Male	− 0.08	− 0.12, − 0.03		.001		− 1.21	− 1.65, − 0.77		< .001
Years of education	0.00	− 0.01, 0.01		.602		0.30	0.22, 0.39		< .001
*APOE* ε4 alleles	− 0.01	− 0.10, 0.08		.808		− 0.41	− 1.31, 0.48		.366
Baseline ICV	NA	NA		NA		NA	NA		NA
SMC	0.05	− 0.00, 0.10		.057		− 0.75	− 1.27, − 0.23		.005
Group									
A − T +	0.06	− 0.01, 0.12		.075		0.16	− 0.46, 0.78		.619
A + T −	− 0.02	− 0.08, 0.04		.520		− 0.31	− 0.87, 0.26		.287
A + T +	− 0.06	− 0.13, 0.01		.104		− 0.34	− 1.06, 0.38		.356
Time	− 0.02	− 0.02, − 0.01		< .001		− 0.01	− 0.10, 0.09		.852
Group × time									
A − T + × time	− 0.01	− 0.02, 0.00		.083		− 0.08	− 0.27, 0.11		.388
A + T − × time	− 0.00	− 0.01, 0.00		.349		− 0.19	− 0.36, − 0.02		.029
A + T + × time	− 0.02	− 0.03, − 0.01		< .001		− 0.59	− 0.80, − 0.37		< .001

*Abbreviations*: *CI* Confidence interval, *ICV* Intracranial volume, *NA* Not applicable, *PACC* Preclinical Alzheimer Cognitive Composite, *SMC* Subjective memory concern

#### Sub-analysis using data from the entire period

Supplementary Figure 2 shows the modeled mean profiles for the MRI measurements and PACC scores including the quadratic term for time and interaction terms between the group and the quadratic term for time in the four groups for the entire period. The likelihood ratio test for the group effect was significant for whole brain (χ2(9) = 32.7; *P* < .001), lateral ventricle (χ2(9) = 81.6; *P* < .001), hippocampus (χ2(9) = 27.5; *P* = .001), cortical thickness (χ2(9) = 36.3; *P* < .001), and PACC scores (χ2(9) = 48.1; *P* < .001). Since these likelihood ratio tests were significant, we determined that including group variables in the statistical model was necessary.

For the whole brain volume and cortical thickness, only the A+T+ group showed statistically significantly greater volume loss and cortical thinning over time than the A−T− group (β = −2.598 cm^3^/year, 95% CI = −4.997 to −0.200, *P* = .034 for the whole brain volume, and β = −0.02 mm/year, 95% CI = −0.04 to −0.01, *P* = .010 for the cortical thickness; Supplementary Figure 2a and Figure 2d and Supplementary Table 4), but no groups showed evidence of acceleration in atrophy rates compared with the reference A−T− group. For the lateral ventricular volume, the A+T− and A+T+ groups showed statistically significantly greater volume expansion than the reference A−T− group (β = 0.634 cm^3^/year, 95% CI = 0.253 to 1.016, *P* = .001 for A+T− vs. A−T−, and β = 1.026 cm^3^/year, 95% CI = 0.547 to 1.505, *P* < .001 for A+T+ vs. A−T−; Supplementary Figure 2b and Supplementary Table 4), but no groups showed evidence of acceleration in atrophy rates compared with the reference A−T− group. For the hippocampal volume, only the A+T− group showed a statistically significant acceleration in atrophy rates compared with the reference A−T− group (β = −0.011 cm^3^/year^2^, 95% CI = −0.019 to −0.004, *P* = .003; Supplementary Figure 2c and Supplementary Table 4). For the PACC score, the A+T− and A+T+ groups showed a statistically significant acceleration in cognitive decline compared with the reference A−T− group (β = −0.05/year^2^, 95% CI = −0.10 to −0.01, *P* = .023 for A+T− vs. A−T−, and β = −0.12/year^2^, 95% CI = −0.18 to −0.06, *P* < .001 for A+T+ vs. A−T−; Supplementary Figure 2e and Supplementary Table 4). In the sensitivity analysis of the data harmonized using longCombat, the A−T+, A+T−, and A+T+ groups all showed an accelerated rate of cortical thinning relative to the reference A−T− group; however, the extent of this acceleration was minor, and differences were notable only beyond the third decimal place (Supplementary Figure 3d and Supplementary Table 5). Regarding the whole brain volume, no group showed a significantly greater reduction over time than the reference A−T− group (Supplementary Figure 3a and Supplementary Table 5). The other results revealed no substantial differences compared with the results using the non-harmonized data.

### Survival analysis for conversion to MCI or dementia

**Fig. 5:**
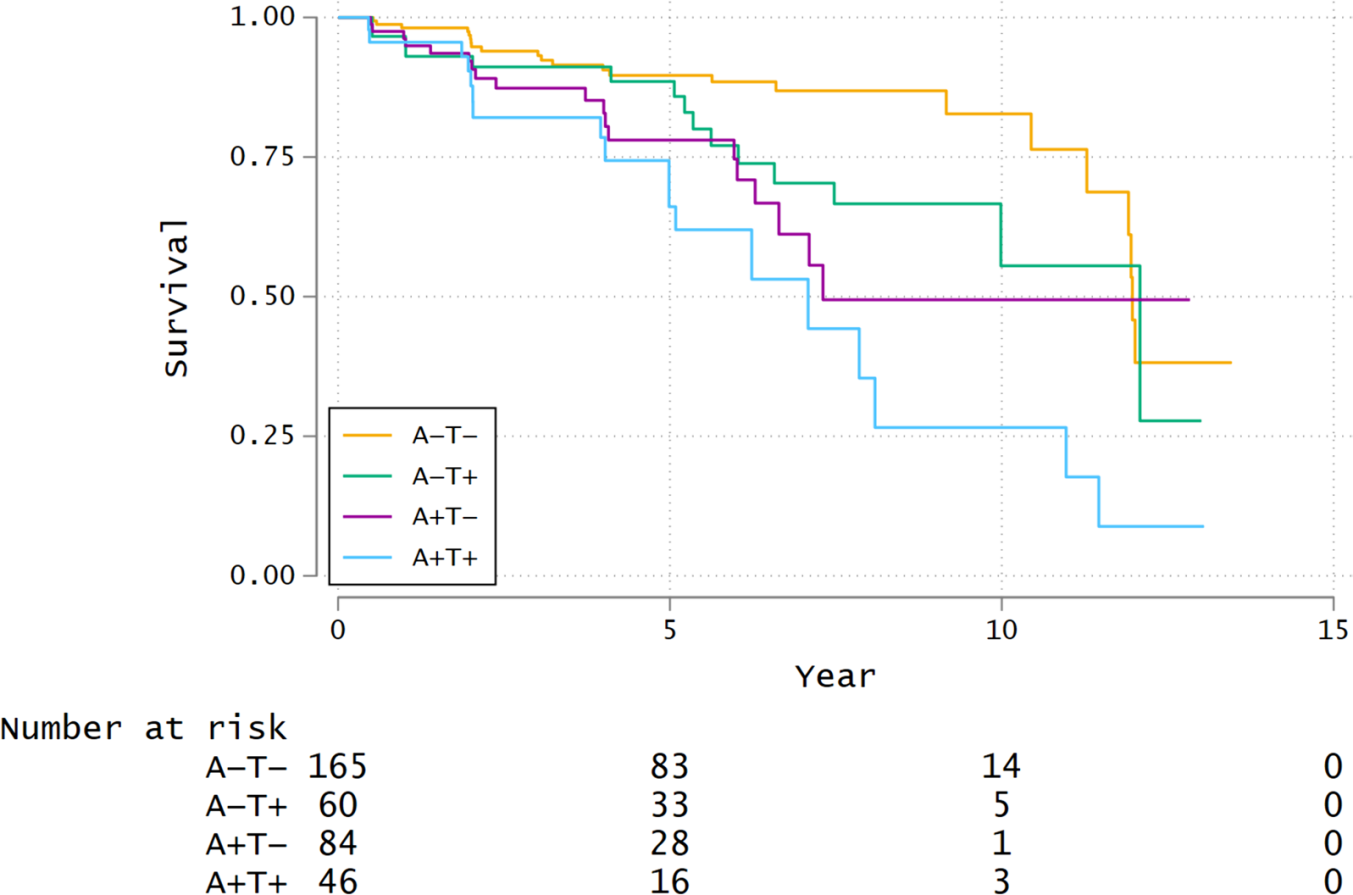
Kaplan–Meier curves on progression to MCI or dementia according to amyloid and tau positivity/negativity. Abbreviations: A −  = amyloid negative; A +  = amyloid positive; T −  = tau negative; T +  = tau positive

Of the 367 participants, 12 had only one observation and were not included in the survival analysis. Therefore, we analyzed 355 participants in the survival analysis. The number of outcome occurrences was 75; of these, 70 were MCI, and 5 were dementia. The mean follow-up time for the two groups was not significantly different at 4.36 years and 8.72 years for the MCI and dementia groups, respectively, (Welch's t-test two-sided *P* = .080). Proportional hazards were confirmed by testing the proportional hazard assumption, which revealed no violation of the assumption (unadjusted model: *P* = .982, adjusted model: *P* = .902). Spearman’s correlation coefficient between the explanatory variables in the adjusted model was at most 0.310 (Group and *APOE* ε4 alleles); therefore, multicollinearity is unlikely. Kaplan–Meier curves showing survival from conversion to MCI or dementia in the four groups are depicted in Fig. 5. The survival curves showed a difference across the four groups (log-rank test, *P* = .001). A Cox proportional hazard analysis revealed that the A + T + and A + T − groups had an increased risk of conversion to MCI or dementia compared with the reference A − T − group in the unadjusted models (hazards ratio = 4.03, 95% CI: 2.17–7.49, *P* < .001 for A + T + vs. A − T − , and hazards ratio = 2.58, 95% CI: 1.39–4.77, *P* = .003 for A + T − vs. A − T − ; Table 3). We also found that the A + T + and A + T − groups had a significantly increased risk of conversion to MCI or dementia in the adjusted models (hazards ratio = 3.35, 95% CI: 1.76–6.39, *P* < .001 for A + T + vs. A − T − , and hazards ratio = 2.38, 95% CI: 1.26–4.48, *P* = .007 for A + T − vs. A − T − ; Table 3). There was no significant difference in survival curves for the A − T + group compared with the A − T − group in the unadjusted and adjusted models.

**Table 3:** Cox proportional hazard ratios for conversion to mild cognitive impairment or dementia

Characteristic	Subgroup	n	Hazards ratio	95% CI		*P*-value
				Lower	Upper	
Unadjusted model						
Group	A − T +	60	1.85	0.96	3.55	.064
	A + T −	84	2.58	1.39	4.77	.003
	A + T +	46	4.03	2.17	7.49	< .001
Adjusted model						
Group	A − T +	60	1.75	0.89	3.44	.106
	A + T −	84	2.38	1.26	4.48	.007
	A + T +	46	3.35	1.76	6.39	< .001
Age			1.06	1.02	1.11	.005
Male			1.54	0.94	2.51	.084
Years of education			0.94	0.87	1.02	.160
*APOE* ε4 alleles			2.19	0.96	5.00	.063
SMC			1.26	0.69	2.29	.446

*Abbreviations*: *CI* Confidence interval, *SMC* Subjective memory concern

## Discussion

### Main findings

The findings of this longitudinal study demonstrated that amyloid positivity (A +) accelerates lateral ventricular expansion, while concurrent positivity for both amyloid and tau (A + T +) precipitates cerebral atrophy affecting the whole brain, hippocampus, and cerebral cortex in the AD-signature regions in cognitively normal elderly individuals. Furthermore, it concomitantly leads to a steep decrement in cognitive performance. Conversely, A − T + does not engender cerebral atrophy or cognitive decline. Additional survival analysis over the entire observation period indicated that A + T + and A + T − augment the subsequent risk for MCI and dementia.

### Interpretation and comparison with previous studies

The results of the main analysis using a generalized linear mixed-effects model in our longitudinal study suggest that A + alone promotes lateral ventricular expansion and that cerebral atrophy becomes more extensive when combined with T + . Desikan et al., Xie et al., and Costoya-Sánchez et al. have already reported accelerated atrophy in subjects with A + T + compared with that in subjects with A − T − in the cerebral cortex and hippocampal subregions [8–10]. Our results are consistent with the findings of these studies. Similar to our study, these studies used the ADNI data, and Costoya-Sánchez et al*.* additionally used data from other longitudinal studies, but their average observation period was approximately two years. In our study, we analyzed data from a longer observation period than these studies and added new longitudinal findings from the whole brain and lateral ventricular volumes and cortical thickness including both the medial and lateral temporal regions, supporting the notion that A + T + accelerates brain atrophy in the regions related to AD.

Notably, unlike the A + T + and A − T + groups, the A + T − group showed expansion of the lateral ventricles at baseline compared with the A − T − group. The reason for this is unclear. Given that lateral ventricular expansion is not specific to AD, it is possible that the A + T − group included a higher proportion of subjects with non-AD causes of lateral ventricular expansion, such as dementia with Lewy bodies [40, 41], or increased white matter hyperintensity volume [42]. During the follow-up period, similar to the A + T + group, the A + T − group exhibited accelerated lateral ventricular expansion compared to the A − T − group. A study on autopsy cases revealed that 50–73% of cases classified as A + T − based on CSF Aβ42 and p-tau181 were found to have AD pathology [43]. For this reason, it should be considered that within the A + T − group in our study, there may be cases in which the T classification determined by p-tau181 could have been a false negative. It is ultimately difficult to ascertain whether the underlying cause of the accelerated lateral ventricular expansion in the A + T − group is due to factors associated with non-AD pathology or whether it is related to amyloid positivity or tau positivity. In addition, the high contrast between the brain parenchyma and CSF may have provided a more reliable longitudinal measurement of the lateral ventricle than those of the other MRI measurements [44], which could have facilitated detection of accelerated lateral ventricular expansion.

With respect to global cognitive performance, our results in the main analysis indicate that A + promotes cognitive decline and that the addition of T + further promotes cognitive decline. There is recent documentation of A + exacerbating cognitive deterioration, and when conjoined with T + , even swifter decline in cognitive function has been demonstrated [7]. These findings are congruent with our own results. In more recent investigations, A + T + has been shown to be associated with an accelerated accumulation of tau in the neocortical region compared with both A − T − and A + T − [10]. Given that the site of tau deposition serves as a predictive indicator for subsequent atrophy within the same cerebral region [45], it is reasonable to anticipate that the presence of A + T + would lead to substantial acceleration of tau deposition and atrophy across the entire cerebral domain. Ultimately, such a process could be poised to induce expedited global cognitive decline.

In contrast, the A − T + group did not show further brain atrophy or decline in cognitive function compared with the reference group, indicating that T + alone does not promote brain atrophy or cognitive decline. Similar to our results, previous studies have also shown that A − T + is not associated with accelerated cognitive decline compared with A − T − [10, 46, 47]. It has been reported that primary age-related tauopathy (PART) [48], a condition probably encompassed within A − T + [49], exhibits an absence of subsequent amyloid elevation, while tau aggregation remains confined to the temporal lobe [10]. Furthermore, the rate of tau accumulation and cognitive decline in PART appears to proceed at a more gradual pace than that observed in individuals with A + T + [10]. Our results support that the A − T + group does not follow the same progressive trajectory as the A + T + group with regard to brain atrophy and cognitive decline.

In the model that incorporated interactions between covariates and time as a sensitivity analysis, we observed significant associations between the baseline age and longitudinal changes in the expansion of the lateral ventricles and cortical thinning. The association between age and the rate of brain atrophy in these regions is supported by our findings and those of other studies [50–52]. Therefore, it is essential to account for the interaction between age and time when conducting longitudinal analyses of volume or cortical thickness in these areas. On the other hand, no significant associations were observed between sex or *APOE* genotype and the rate of brain atrophy. There are conflicting reports regarding the association between sex and brain atrophy rates [50, 52]. Although some studies have suggested an association between the *APOE* genotype and atrophy rates in the medial temporal regions [52, 53], our results are not in accordance with these findings. These conflicting findings highlight the need for further research to further elucidate this relationship.

Additional sub-analyses using a generalized linear mixed-effects model with linear and quadratic terms for time and their interaction with the group using data from the entire period showed similar results to those of the main analysis using only the linear term for time with respect to whole brain and lateral ventricle volumes and cortical thickness in the AD-signature regions. While the A + T − and A + T + groups showed greater acceleration of decline (quadratic effect) than the A − T − group in terms of the PACC scores, only the A + T − group showed greater acceleration of volume loss in the hippocampus. The reason why the A + T + group did not show a significantly greater hippocampal volume reduction with respect to the linear and/or quadratic terms of time than the A − T − group is unclear. However, one possibility is that the data after 7.5 years from baseline may have been affected by outliers due to few observations.

In this study, the structural MRI scans were longitudinally acquired from scanners from different sites, scanner manufacturers, and magnetic field strengths of 1.5 T and 3 T, which may have introduced undesirable, nonbiological technical variations in the inter- and/or intrasubject measurements obtained from them [54–56]. Image harmonization techniques, such as longitudinal ComBat, may minimize such non-biological sources of variances among different scanners in this multisite longitudinal study [37, 57]. Thus, we employed longitudinal ComBat (via longCombat package in R) to harmonize the MRI measurements, aiming to mitigate the non-biological variability for the sensitivity analyses. In the sub-analysis that included the quadratic term for time and interaction terms between group and the quadratic term for time across the four groups for the entire period, none of the groups demonstrated a significant reduction in longitudinal whole brain volume compared with the reference A − T − group. This observation could potentially be explained by the regulation of Type 1 errors through longitudinal ComBat, as indicated by Beer et al. [37]. Nonetheless, other findings showed negligible discrepancies compared with the outcomes derived from the examination of data that had not been harmonized.

In our results of the survival analysis, only the A + T + and A + T − groups showed a statistically significant increased risk of progression to MCI or dementia compared with the reference A − T − group. Prior studies have demonstrated that A + T + is associated with a higher risk of disease progression in AD than A − T − [6, 7], and our results are consistent with those findings. Populations with a combination of A + and T + are at higher risk for disease progression in AD during the preclinical stage; therefore, therapeutic intervention in these populations may effectively inhibit disease progression. The A + T − group also demonstrated an increased risk of disease progression to MCI or dementia compared with the reference group. As mentioned earlier, it is plausible that among cases classified as A + T − , there were instances where the determination of tau pathology via CSF p-tau181 resulted in false negatives. This potential misclassification might have influenced the observed risk elevation within this group.

Treatments for MCI and mild dementia using anti-amyloid antibodies such as lecanemab and donanemab have been shown to slow down cognitive decline in Phase 3 clinical trials, but they have not demonstrated the ability to halt cognitive decline [1, 2]. Our study has shown that elderly individuals with preclinical AD classified as A + T + experience accelerated brain atrophy and cognitive decline compared with those classified as A − T − . This suggests that proactive treatment in such elderly individuals with anti-amyloid antibodies, as in the AHEAD trial or TRAILBLAZER-ALZ3 (NCT05026866), or treatment targeting tau might help in preventing the progression of brain atrophy and cognitive decline. However, one meta-analysis indicated that certain anti-amyloid therapies including some anti-amyloid antibodies and secretase inhibitors may accelerate brain atrophy [58]. It remains unclear whether this atrophy is a result of these treatments exacerbating neurodegeneration or due to other causes; thus, further research is needed.

## Limitations of the study

Our study has four limitations:

1. We had a small number of observations after 7.5 years. Therefore, analyses of brain morphology and cognitive function for the entire period including after 7.5 years may have been influenced by outliers. Survival analyses also may have been affected by selection bias due to dropouts after 7.5 years. It is anticipated that at least half of the ADNI3 participants will continue to participate in ADNI4 [59], which may make it possible to analyze observational data over a longer period of time.
2. In our analysis, we compared linear and quadratic models. Despite the better fit of the quadratic models as indicated by their lower AIC values, we selected a linear model due to its simplicity and clinical interpretability. This decision balanced model complexity with interpretability, which is crucial for clinical application. However, we acknowledge that quadratic models may capture data nuances more accurately; thus, further exploration in future studies is warranted.
3. We used CSF Aβ42 to evaluate A + /– instead of the concentration ratio of Aβ42 to Aβ40 (Aβ42/40 ratio) because there were numerous cases in which Aβ40 was not measured in the ADNI data. The Aβ42/40 ratio is more accurate in diagnosing patients with AD than Aβ42 alone; therefore, Hansson et al. advocate using the former when analyzing CSF AD biomarkers [60]. Future studies should be able to discriminate A + /– with higher accuracy using the Aβ42/40 ratio.
4. We used CSF p-tau181 to evaluate T + /–. While CSF p-tau181 is a biomarker for T that represents changes in tau metabolism in the AT(N) system [5], elevated CSF p-tau181 levels are more strongly associated with cerebral amyloidosis than with neurofibrillary tangles [61]. Due to the potential of CSF p-tau205 and CSF microtubule-binding region (MTBR) of tau containing the residue 243 (MTBR-tau243) as indicators of tau tangles [62], employing T defined by Tau-PET, CSF p-tau205, or CSF MTBR-tau243 would allow for a longitudinal study of cognitively healthy individuals in a condition that better reflects the pathological changes of amyloid plaques and neurofibrillary tangles.

## Conclusions

Within the healthy elderly population, A + , when combined with T + , exacerbates cerebral atrophy in the regions related to AD, leading to accelerated disease progression. Notably, in isolation, T + does not provoke cerebral atrophy, cognitive decline, or disease progression. The implementation of therapeutic intervention in cognitively normal individuals with A + T + may serve as a pivotal strategy in forestalling subsequent cerebral atrophy, cognitive deterioration, and disease progression.

### Supplementary Information

**Supplementary Material 1.**

**Supplementary Material 2.**

## Data Availability

The ADNI data is publicly available on its database for approved individuals (https://adni.loni.usc.edu/). The processed data in this study are not publicly available because of space limitations but are available from the corresponding author on reasonable request.

## Abbreviations

Aβ42: Amyloid-beta 42
AD: Alzheimer's disease
ADNI: Alzheimer's Disease Neuroimaging Initiative
AIC: Akaike Information Criterion
APOE: Apolipoprotein E
A −: Amyloid negative
A +: Amyloid positive
CDR: Clinical Dementia Rating
CSF: Cerebrospinal fluid
DBC: Differential bias correction
DiReCT: Diffeomorphic registration-based cortical thickness
DL: Deep learning-based neuroanatomy segmentation and cortex parcellation
KN-BSI: K-means normalized boundary shift integral
LOESS: Locally estimated scatterplot smoothing
MCI: Mild cognitive impairment
MMSE: Mini-Mental State Examination
MRI: Magnetic resonance imaging
MTBR-tau243: Microtubule-binding region of tau containing the residue 243
PACC: Preclinical Alzheimer Cognitive Composite
PART: Primary age-related tauopathy
PET: Positron emission tomography
p-tau181: Phosphorylated tau 181
T −: Tau negative
T +: Tau positive
WMS-R: Wechsler Memory Scale-Revised
