# Supplementary material for: Impact of amyloid and tau positivity on longitudinal brain atrophy in cognitively normal individuals
Source: Alzheimers Res Ther. 2024 Apr 10;16:77. doi: 10.1186/s13195-024-01450-7 (PMC11005141; doi:10.1186/s13195-024-01450-7)
Supplement: Supplementary file 1 — Supplementary Material 1. [file 13195_2024_1450_MOESM1_ESM.docx]

Supplementary Figure 1. Changes in longitudinal MRI measurements (harmonized using longCombat) by amyloid and tau positivity/negativity classification over 7.5 years.

a) - d) Trajectories of modeled mean profiles of whole brain volume, lateral ventricular volume, hippocampal volume, and cortical thickness based on a generalized linear mixed-effects model and 95% confidence bands. The models were controlled for baseline age, *APOE* ε4 status, sex, number of years of education, and baseline intracranial volume (only for the volumetric measures).

Abbreviations: A− = amyloid negative; A+ = amyloid positive; T− = tau negative; T+ = tau positive.

Supplementary Figure 2. Changes in longitudinal MRI measurements and cognitive performance by amyloid and tau positivity/negativity classification for the entire period.

a) - e) Trajectories of modeled mean profiles of whole brain volume, lateral ventricular volume, hippocampal volume, cortical thickness, and PACC scores based on a generalized linear mixed-effects model and 95% confidence bands. The models were controlled for baseline age, *APOE* ε4 status, sex, number of years of education, and baseline intracranial volume (only for the volumetric measures).

Abbreviations: A− = amyloid negative; A+ = amyloid positive; PACC = Preclinical Alzheimer Cognitive Composite; T− = tau negative; T+ = tau positive.

Supplementary Figure 3. Changes in longitudinal MRI measurements (harmonized using longCombat) by amyloid and tau positivity/negativity classification for the entire period.

a) - d) Trajectories of modeled mean profiles of whole brain volume, lateral ventricular volume, hippocampal volume, and cortical thickness based on a generalized linear mixed-effects model and 95% confidence bands. The models were controlled for baseline age, *APOE* ε4 status, sex, number of years of education, and baseline intracranial volume (only for the volumetric measures).

Abbreviations: A− = amyloid negative; A+ = amyloid positive; T− = tau negative; T+ = tau positive.
