# Supplementary material for: Impact of amyloid and tau positivity on longitudinal brain atrophy in cognitively normal individuals
Source: Alzheimers Res Ther. 2024 Apr 10;16:77. doi: 10.1186/s13195-024-01450-7 (PMC11005141; doi:10.1186/s13195-024-01450-7)
Supplement: Supplementary file 2 — Supplementary Material 2. [file 13195_2024_1450_MOESM2_ESM.docx]

Supplementary Table 1. Summary of AIC comparison between linear and quadratic models in the main analysis.

|  | Whole brain | Lateral ventricle | Hippocampus | Cortical thickness | PACC |
| --- | --- | --- | --- | --- | --- |
| Liner model | 15524.78 | 8894.20 | 635.95 | −3372.33 | 7527.87 |
| Quadratic model | 15338.22 | 8747.39 | 251.47 | −3408.40 | 7481.30 |

Abbreviations: AIC = Akaike Information Criterion.

Supplementary Table 2. Summary of a generalized linear mixed-effects model for serial structural MRI and cognitive performance measures from baseline to 7.5 years with additional covariates and time interactions.

|  | Whole brain | | | Lateral ventricle | | | Hippocampus | | |
| --- | --- | --- | --- | --- | --- | --- | --- | --- | --- |
|  | β | 95% CI | *P*-value | β | 95% CI | *P*-value | β | 95% CI | *P*-value |
|  |  | Lower, Upper |  |  | Lower, Upper |  |  | Lower, Upper |  |
| Intercept | 1369.409 | 1297.636, 1441.183 | < .001 | −37.499 | −59.500, −15.498 | .001 | 11.799 | 10.673, 12.925 | < .001 |
| Age | −4.233 | −5.103, −3.363 | < .001 | 0.977 | 0.710, 1.243 | < .001 | −0.051 | −0.064, −0.037 | < .001 |
| Male | 16.516 | 4.728, 28.305 | .006 | −1.588 | −5.203, 2.027 | .389 | 0.099 | −0.086, 0.284 | .294 |
| Years of education | −0.341 | −2.255, 1.574 | .727 | 0.117 | −0.470, 0.704 | .696 | −0.039 | −0.069, −0.009 | .011 |
| *APOE* ε4 alleles | 16.424 | −3.904, 36.753 | .113 | −9.938 | −16.170, −3.706 | .002 | 0.091 | −0.228, 0.410 | .574 |
| Baseline ICV | 0.539 | 0.502, 0.575 | < .001 | 0.055 | 0.044, 0.066 | < .001 | 0.003 | 0.002, 0.003 | < .001 |
| SMC | 14.494 | 3.125, 25.863 | .012 | 2.545 | −0.937, 6.027 | .152 | 0.069 | −0.110, 0.247 | .452 |
| Group |  |  |  |  |  |  |  |  |  |
| A−T+ | 5.702 | −8.322, 19.726 | .426 | −3.911 | −8.211, 0.388 | .075 | −0.028 | −0.248, 0.192 | .801 |
| A+T− | −16.608 | −29.371, −3.845 | .011 | 13.425 | 9.512, 17.338 | < .001 | −0.169 | −0.369, 0.032 | .099 |
| A+T+ | −2.075 | −18.337, 14.188 | .803 | 3.758 | −1.228, 8.743 | .140 | −0.057 | −0.312, 0.198 | .662 |
| Time | −7.009 | −12.885, −1.133 | .019 | −0.852 | −2.458, 0.753 | .298 | −0.056 | −0.187, 0.075 | .400 |
| Age × time | 0.013 | −0.057, 0.083 | .720 | 0.031 | 0.011, 0.050 | .002 | −0.001 | −0.002, 0.001 | .272 |
| Male × time | −0.205 | −1.153, 0.742 | .671 | 0.052 | −0.209, 0.314 | .694 | 0.010 | −0.011, 0.031 | .338 |
| Years of education × time | 0.015 | −0.136, 0.166 | .846 | −0.011 | −0.053, 0.031 | .599 | 0.002 | −0.002, 0.005 | .315 |
| *APOE* ε4 alleles × time | −0.108 | −1.755, 1.539 | .897 | −0.192 | −0.646, 0.262 | .408 | −0.029 | −0.066, 0.008 | .119 |
| Baseline ICV × time | −0.004 | −0.007, −0.001 | .005 | 0.002 | 0.001, 0.003 | < .001 | 0.000 | 0.000, 0.000 | .459 |
| SMC × time | 0.468 | −0.489, 1.425 | .338 | 0.045 | −0.214, 0.305 | .731 | −0.004 | −0.025, 0.017 | .707 |
| Group × time |  |  |  |  |  |  |  |  |  |
| A−T+ × time | −0.538 | −1.661, 0.584 | .347 | −0.046 | −0.357, 0.266 | .775 | −0.021 | −0.046, 0.004 | .092 |
| A+T− × time | −0.473 | −1.513, 0.567 | .373 | 0.746 | 0.460, 1.031 | < .001 | −0.011 | −0.034, 0.013 | .369 |
| A+T+ × time | −2.735 | −4.051, −1.419 | < .001 | 0.794 | 0.430, 1.158 | < .001 | −0.049 | −0.078, −0.019 | .001 |

|  | Cortical thickness | | | PACC | | |
| --- | --- | --- | --- | --- | --- | --- |
|  | β | 95% CI | *P*-value | β | 95% CI | *P*-value |
|  |  | Lower, Upper |  |  | Lower, Upper |  |
| Intercept | 4.09 | 3.77, 4.41 | < .001 | 5.092 | 1.921, 8.262 | .002 |
| Age | −0.02 | −0.02, −0.01 | < .001 | −0.125 | −0.163, −0.086 | < .001 |
| Male | −0.08 | −0.13, −0.04 | .001 | −1.208 | −1.653, −0.763 | < .001 |
| Years of education | 0.00 | −0.01, 0.01 | .566 | 0.308 | 0.224, 0.392 | < .001 |
| *APOE* ε4 alleles | −0.01 | −0.10, 0.08 | .842 | −0.376 | −1.277, 0.525 | .414 |
| Baseline ICV | NA | NA | NA | NA | NA | NA |
| SMC | 0.05 | −0.01, 0.10 | .083 | −0.787 | −1.311, −0.263 | .003 |
| Group |  |  |  |  |  |  |
| A−T+ | 0.06 | −0.01, 0.12 | .080 | 0.154 | −0.464, 0.773 | .624 |
| A+T− | −0.02 | −0.08, 0.04 | .492 | −0.324 | −0.889, 0.240 | .260 |
| A+T+ | −0.06 | −0.14, 0.01 | .095 | −0.358 | −1.077, 0.360 | .328 |
| Time | 0.03 | −0.02, 0.08 | .223 | 0.984 | −0.009, 1.977 | .052 |
| Age × time | −0.00 | −0.00, −0.00 | .042 | −0.013 | −0.025, −0.001 | .036 |
| Male × time | 0.00 | −0.00, 0.01 | .274 | 0.006 | −0.131, 0.144 | .926 |
| Years of education × time | 0.00 | −0.00, 0.00 | .567 | −0.005 | −0.030, 0.020 | .695 |
| *APOE* ε4 alleles × time | −0.00 | −0.02, 0.01 | .559 | −0.105 | −0.384, 0.174 | .463 |
| Baseline ICV × time | NA | NA | NA | NA | NA | NA |
| SMC × time | 0.01 | 0.00, 0.02 | .043 | 0.076 | −0.089, 0.242 | .366 |
| Group × time |  |  |  |  |  |  |
| A−T+ × time | −0.01 | −0.02, 0.00 | .215 | −0.047 | −0.236, 0.143 | .628 |
| A+T− × time | −0.00 | −0.01, 0.01 | .688 | −0.152 | −0.328, 0.024 | .091 |
| A+T+ × time | −0.02 | −0.03, −0.01 | < .001 | −0.526 | −0.747, −0.305 | < .001 |

Abbreviations: CI = confidence interval; ICV = intracranial volume; NA = not applicable; PACC = Preclinical Alzheimer Cognitive Composite; SMC = subjective memory concern.

Supplementary Table 3. Summary of a generalized linear mixed-effects model in the primary analysis for serial structural MRI measures (harmonized using longCombat) from baseline to 7.5 years.

|  | Whole brain | | | Lateral ventricle | | | Hippocampus | | |
| --- | --- | --- | --- | --- | --- | --- | --- | --- | --- |
|  | β | 95% CI | *P*-value | β | 95% CI | *P*-value | β | 95% CI | *P*-value |
|  |  | Lower, Upper |  |  | Lower, Upper |  |  | Lower, Upper |  |
| Intercept | 1326.649 | 1269.528, 1383.770 | < .001 | −14.824 | −31.906, 2.257 | .089 | 11.556 | 10.568, 12.544 | < .001 |
| Age | −3.846 | −4.535, −3.157 | < .001 | 0.739 | 0.534, 0.944 | < .001 | −0.050 | −0.062, −0.038 | < .001 |
| Male | 14.701 | 5.366, 24.036 | .002 | −3.575 | −6.351, −0.800 | .012 | 0.180 | 0.019, 0.342 | .029 |
| Years of education | −0.084 | −1.600, 1.433 | .914 | 0.033 | −0.417, 0.483 | .886 | −0.039 | −0.065, −0.013 | .003 |
| *APOE* ε4 alleles | 7.369 | −8.734, 24.473 | .370 | −6.675 | −11.476, −1.874 | .006 | 0.058 | −0.220, 0.337 | .681 |
| Baseline ICV | 0.565 | 0.536, 0.594 | < .001 | 0.052 | 0.044, 0.061 | < .001 | 0.003 | 0.002, 0.003 | < .001 |
| SMC | −1.107 | −10.125, 7.911 | .810 | 2.676 | −0.027, 5.380 | .052 | 0.096 | −0.060, 0.252 | < .001 |
| Group |  |  |  |  |  |  |  |  |  |
| A−T+ | 0.446 | −10.663, 11.556 | .937 | −2.082 | −5.855, 1.690 | .279 | −0.147 | −3.40, 0.047 | .137 |
| A+T− | −10.662 | −20.772, −0.552 | .039 | 11.895 | 8.472, 15.317 | < .001 | −0.182 | −0.358, −0.006 | .043 |
| A+T+ | 6.568 | −6.315, 19.451 | .318 | 4.987 | 0.657, 9.318 | .024 | −0.166 | −0.391, 0.058 | .146 |
| Time | −5.932 | −6.545, −5.319 | < .001 | 1.247 | 1.073, 1.420 | < .001 | −0.092 | −0.105, −0.078 | < .001 |
| Group × time |  |  |  |  |  |  |  |  |  |
| A−T+ × time | −0.354 | −1.556, 0.847 | .563 | −0.056 | −0.393, 0.281 | .745 | −0.022 | −0.049, 0.005 | .107 |
| A+T− × time | −0.205 | −1.304, 0.894 | .714 | 0.743 | 0.439, 1.048 | < .001 | −0.011 | −0.035, 0.014 | .387 |
| A+T+ × time | −2.822 | −4.193, −1.451 | < .001 | 0.894 | 0.513, 1.274 | < .001 | −0.052 | −0.083, −0.022 | .001 |

(Table continues on next page)

|  | Cortical thickness | | |
| --- | --- | --- | --- |
|  | β | 95% CI | *P*-value |
|  |  | Lower, Upper |  |
| Intercept | 4.16 | 3.91, 4.41 | < .001 |
| Age | −0.02 | −0.02, −0.01 | < .001 |
| Male | −0.08 | −0.11, −0.04 | < .001 |
| Years of education | −0.00 | −0.01, 0.00 | .260 |
| *APOE* ε4 alleles | −0.04 | −0.11, 0.04 | .328 |
| Baseline ICV | NA | NA | NA |
| SMC | −0.03 | −0.07, 0.01 | .194 |
| Group |  |  |  |
| A−T+ | 0.08 | 0.03, 0.13 | .001 |
| A+T− | 0.01 | −0.04, 0.05 | .831 |
| A+T+ | −0.02 | −0.08, 0.04 | .480 |
| Time | −0.02 | −0.02, −0.01 | < .001 |
| Group × time |  |  |  |
| A−T+ × time | −0.01 | −0.02, 0.00 | .062 |
| A+T− × time | −0.01 | −0.01, 0.00 | .213 |
| A+T+ × time | −0.02 | −0.03, −0.01 | < .001 |

Abbreviations: CI = confidence interval; ICV = intracranial volume; NA = not applicable; SMC = subjective memory concern.

Supplementary Table 4. Summary of a generalized linear mixed-effects model for serial structural MRI and cognitive performance measures from the entire period.

|  | Whole brain | | | Lateral ventricle | | | Hippocampus | | |
| --- | --- | --- | --- | --- | --- | --- | --- | --- | --- |
|  | β | 95% CI | *P*-value | β | 95% CI | *P*-value | β | 95% CI | *P*-value |
|  |  | Lower, Upper |  |  | Lower, Upper |  |  | Lower, Upper |  |
| Intercept | 1356.446 | 1284.536, 1428.355 | < .001 | −23.816 | −43.338, −4.295 | .017 | 11.857 | 10.731, 12.982 | < .001 |
| Age | −4.264 | −5.131, −3.396 | < .001 | 0.800 | 0.566, 1.035 | < .001 | −0.053 | −0.066, −0.039 | < .001 |
| Male | 16.758 | 5.005, 28.511 | .005 | −2.020 | −5.191, 1.151 | .212 | 0.104 | −0.080, 0.288 | .268 |
| Years of education | −0.353 | −2.261, 1.556 | .717 | 0.196 | −0.318, 0.710 | .455 | −0.039 | −0.069, −0.009 | .011 |
| *APOE* ε4 alleles | 16.430 | −3.842, 36.703 | .112 | −8.205 | −13.690, −2.720 | .003 | 0.089 | −0.228, 0.407 | .582 |
| Baseline ICV | 0.542 | 0.506, 0.578 | < .001 | 0.042 | 0.032, 0.051 | < .001 | 0.003 | 0.002, 0.003 | < .001 |
| SMC | 13.805 | 2.468, 25.141 | .017 | 2.046 | −1.040, 5.133 | .194 | 0.097 | −0.080, 0.275 | .283 |
| Group |  |  |  |  |  |  |  |  |  |
| A−T+ | 5.620 | −8.390, 19.630 | .432 | −3.801 | −8.133, 0.531 | .086 | −0.034 | −0.253, 0.185 | .759 |
| A+T− | −17.356 | −30.107, −4.606 | .008 | 13.468 | 9.538, 17.397 | < .001 | −0.197 | −0.396, 0.003 | .053 |
| A+T+ | −2.038 | −18.284, 14.208 | .806 | 4.042 | −0.929, 9.014 | .111 | −0.071 | −0.325, 0.183 | .585 |
| Time | −8.130 | −9.181, −7.078 | < .001 | 1.351 | 1.135, 1.566 | < .001 | −0.145 | −0.172, −0.119 | < .001 |
| Group × time |  |  |  |  |  |  |  |  |  |
| A−T+ × time | 0.171 | −1.857, 2.198 | .869 | −0.155 | −0.571, 0.261 | .465 | 0.005 | −0.047, 0.056 | .859 |
| A+T− × time | 0.569 | −1.325, 2.463 | .556 | 0.634 | 0.253, 1.016 | .001 | 0.045 | −0.002, 0.093 | .061 |
| A+T+ × time | −2.598 | −4.997, −0.200 | .034 | 1.026 | 0.547, 1.505 | < .001 | −0.016 | −0.076, 0.044 | .597 |
| Time^2^ | 0.405 | 0.278, 0.531 | < .001 | −0.020 | −0.040, 0.000 | .053 | 0.009 | 0.005, 0.013 | < .001 |
| Group × time^2^ |  |  |  |  |  |  |  |  |  |
| A−T+ × time^2^ | −0.076 | −0.313, 0.161 | .530 | 0.024 | −0.014, 0.062 | .212 | −0.005 | −0.013, 0.003 | .221 |
| A+T− × time^2^ | −0.216 | −0.455, 0.023 | .077 | 0.023 | −0.016, 0.061 | .245 | −0.011 | −0.019, −0.004 | .003 |
| A+T+ × time^2^ | −0.017 | −0.328, 0.295 | .917 | −0.031 | −0.081, 0.019 | .223 | −0.007 | −0.016, 0.003 | .153 |

|  | Cortical thickness | | | PACC | | |
| --- | --- | --- | --- | --- | --- | --- |
|  | β | 95% CI | *P*-value | β | 95% CI | *P*-value |
|  |  | Lower, Upper |  |  | Lower, Upper |  |
| Intercept | 4.17 | 3.86, 4.49 | < .001 | 6.33 | 3.25, 9.41 | < .001 |
| Age | −0.02 | −0.02, −0.01 | < .001 | −0.12 | −0.16, −0.09 | < .001 |
| Male | −0.08 | −0.12, −0.03 | .001 | −1.22 | −1.65, −0.78 | < .001 |
| Years of education | 0.00 | −0.01, 0.01 | .670 | 0.30 | 0.21, 0.38 | < .001 |
| *APOE* ε4 alleles | −0.01 | −0.10, 0.08 | .810 | −0.39 | −1.27, 0.49 | .388 |
| Baseline ICV | NA | NA | NA | NA | NA | NA |
| SMC | 0.05 | −0.00, 0.10 | .064 | −0.67 | −1.18, −0.16 | .010 |
| Group |  |  |  |  |  |  |
| A−T+ | 0.05 | −0.01, 0.12 | .084 | −0.03 | −0.64, 0.58 | .924 |
| A+T− | −0.02 | −0.08, 0.03 | .438 | −0.45 | −1.02, 0.11 | .116 |
| A+T+ | −0.06 | −0.13, 0.01 | .094 | −0.72 | −1.43, −0.00 | .049 |
| Time | −0.02 | −0.03, −0.02 | < .001 | 0.16 | −0.00, 0.33 | .055 |
| Group × time |  |  |  |  |  |  |
| A−T+ × time | −0.01 | −0.02, 0.01 | .350 | 0.18 | −0.14, 0.49 | .268 |
| A+T− × time | 0.00 | −0.01, 0.02 | .572 | 0.09 | −0.22, 0.40 | .563 |
| A+T+ × time | −0.02 | −0.04, −0.01 | .010 | 0.07 | −0.31, 0.46 | .709 |
| Time^2^ | 0.00 | 0.00, 0.00 | .001 | −0.03 | −0.05, −0.01 | .011 |
| Group × time^2^ |  |  |  |  |  |  |
| A−T+ × time^2^ | −0.00 | −0.00, 0.00 | .682 | −0.04 | −0.08, 0.01 | .086 |
| A+T− × time^2^ | −0.00 | −0.00, 0.00 | .064 | −0.05 | −0.10, −0.01 | .023 |
| A+T+ × time^2^ | −0.00 | −0.00, 0.00 | .635 | −0.12 | −0.18, −0.06 | < .001 |

Abbreviations: CI = confidence interval; ICV = intracranial volume; NA = not applicable; PACC = Preclinical Alzheimer Cognitive Composite; SMC = subjective memory concern.

Supplementary Table 5. Summary of a generalized linear mixed-effects model for serial structural MRI measures (harmonized using longCombat) from the entire period.

|  | Whole brain | | | Lateral ventricle | | | Hippocampus | | |
| --- | --- | --- | --- | --- | --- | --- | --- | --- | --- |
|  | β | 95% CI | *P*-value | β | 95% CI | *P*-value | β | 95% CI | *P*-value |
|  |  | Lower, Upper |  |  | Lower, Upper |  |  | Lower, Upper |  |
| Intercept | 1325.552 | 1269.512, 1381.593 | < .001 | −17.265 | −33.978, −0.552 | .043 | 11.896 | 10.9.23, 12.869 | < .001 |
| Age | −3.875 | −4.551, −3.200 | < .001 | 0.779 | 0.579, 0.980 | < .001 | −0.053 | −0.065, −0.042 | < .001 |
| Male | 12.926 | 3.781, 22.072 | .006 | −3.618 | −6.329, −0.907 | .009 | 0.188 | 0.029, 0.347 | .021 |
| Years of education | 0.354 | −1.132, 1.840 | .640 | 0.020 | −0.419, 0.459 | .930 | −0.043 | −0.069, −0.017 | .001 |
| *APOE* ε4 alleles | 9.517 | −6.276, 25.309 | .238 | −6.255 | −10.948, −1.563 | .009 | 0.035 | −0.240, 0.309 | .805 |
| Baseline ICV | 0.575 | 0.547, 0.603 | < .001 | 0.051 | 0.043, 0.060 | < .001 | 0.003 | 0.002, 0.003 | < .001 |
| SMC | 0.252 | −8.595, 9.100 | .955 | 1.571 | −1.072, 4.215 | .244 | 0.021 | −0.132, 0.175 | .785 |
| Group |  |  |  |  |  |  |  |  |  |
| A−T+ | −1.237 | −12.198, 9.724 | .825 | −2.212 | −5.943, 1.519 | .245 | −0.127 | −0.317, 0.062 | .188 |
| A+T− | −9.403 | −19.378, 0.573 | .065 | 11.428 | 8.044, 14.812 | < .001 | −0.197 | −0.369, −0.024 | .025 |
| A+T+ | 3.210 | −9.500, 15.919 | .621 | 5.121 | 0.841, 9.401 | .019 | −0.123 | −0.343, 0.097 | .273 |
| Time | −7.971 | −9.108, −6.835 | < .001 | 1.369 | 1.137, 1.602 | < .001 | −0.143 | −0.171, −0.144 | < .001 |
| Group × time |  |  |  |  |  |  |  |  |  |
| A−T+ × time | 0.173 | −2.016, 2.362 | .877 | −0.133 | −0.581, 0.316 | .562 | 0.010 | −0.045, 0.065 | .710 |
| A+T− × time | 0.237 | −1.821, 2.294 | .822 | 0.655 | 0.242, 1.068 | .002 | 0.046 | −0.006, 0.097 | .081 |
| A+T+ × time | −2.379 | −4.983, 0.226 | .073 | 1.009 | 0.490, 1.528 | < .001 | −0.016 | −0.081, 0.049 | .634 |
| Time^2^ | 0.382 | 0.245, 0.518 | < .001 | −0.023 | −0.046, −0.001 | .043 | 0.009 | 0.005, 0.013 | < .001 |
| Group × time^2^ |  |  |  |  |  |  |  |  |  |
| A−T+ × time^2^ | −0.091 | −0.346, 0.165 | .487 | 0.019 | −0.024, 0.061 | .384 | −0.006 | −0.014, 0.002 | .159 |
| A+T− × time^2^ | −0.156 | −0.416, 0.105 | .241 | 0.019 | −0.024, 0.062 | .393 | −0.012 | −0.020, −0.004 | .004 |
| A+T+ × time^2^ | −0.080 | −0.419, 0.259 | .644 | −0.028 | −0.084, 0.028 | .335 | −0.007 | −0.017, 0.004 | .201 |

|  | Cortical thickness | | |
| --- | --- | --- | --- |
|  | β | 95% CI | *P*-value |
|  |  | Lower, Upper |  |
| Intercept | 4.16 | 3.91, 4.41 | <.001 |
| Age | −0.02 | −0.02, −0.01 | < .001 |
| Male | −0.08 | −0.11, −0.04 | < .001 |
| Years of education | −0.00 | −0.01, 0.00 | .263 |
| *APOE* ε4 alleles | −0.04 | −0.11, 0.04 | .338 |
| Baseline ICV | NA | NA | NA |
| SMC | −0.03 | −0.07, 0.01 | .164 |
| Group |  |  |  |
| A−T+ | 0.08 | 0.03, 0.13 | .002 |
| A+T− | 0.00 | −0.04, 0.05 | .980 |
| A+T+ | −0.02 | −0.08, 0.03 | .409 |
| Time | −0.02 | −0.03, −0.02 | < .001 |
| Group × time |  |  |  |
| A−T+ × time | −0.00 | −0.02, 0.01 | .604 |
| A+T− × time | 0.00 | −0.01, 0.02 | .449 |
| A+T+ × time | −0.02 | −0.03, −0.00 | .023 |
| Time^2^ | 0.00 | 0.00, 0.00 | .001 |
| Group × time^2^ |  |  |  |
| A−T+ × time^2^ | −0.00 | −0.00, −0.00 | .049 |
| A+T− × time^2^ | −0.00 | −0.00, −0.00 | .003 |
| A+T+ × time^2^ | −0.00 | −0.00, −0.00 | .031 |

Abbreviations: CI = confidence interval; ICV = intracranial volume; NA = not applicable; SMC = subjective memory concern.
